# Imaging Interface and Particle Size Effects by In Situ Correlative Microscopy of a Catalytic Reaction

**DOI:** 10.1021/acscatal.3c00060

**Published:** 2023-05-23

**Authors:** Philipp Winkler, Maximilian Raab, Johannes Zeininger, Lea M. Rois, Yuri Suchorski, Michael Stöger-Pollach, Matteo Amati, Rahul Parmar, Luca Gregoratti, Günther Rupprechter

**Affiliations:** † Institute of Materials Chemistry, TU Wien, Getreidemarkt 9, Vienna 1060, Austria; ‡ University Service Center for Transmission Electron Microscopy, TU Wien, Wiedner Hauptstraße 8-10, Vienna 1040, Austria; § Elettra-Sincrotrone Trieste S.C.p.A., SS 14 km 163.5 in AREA Science Park, Trieste 34149, Italy

**Keywords:** interface effects, size effect, correlative microscopy, photoemission electron microscopy, scanning photoemission microscopy, micro-kinetic modeling, catalytic hydrogen oxidation

## Abstract

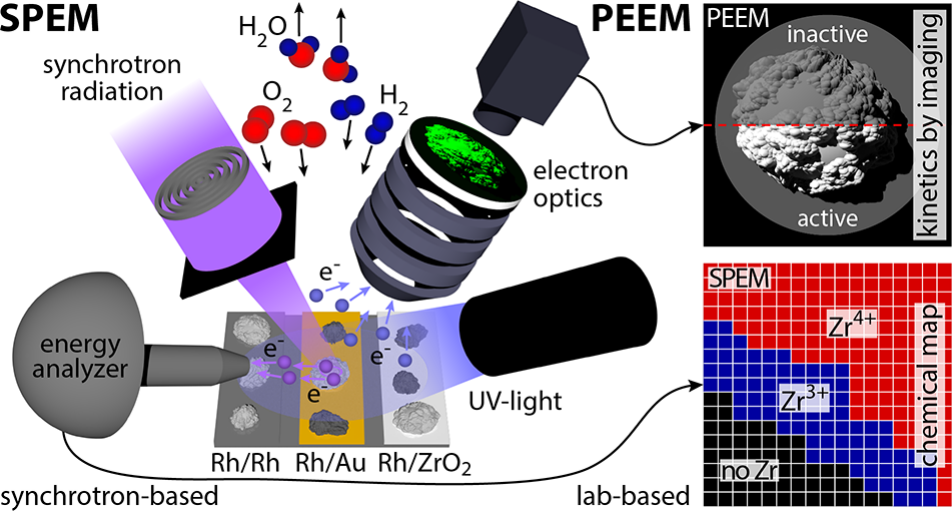

The catalytic behavior of Rh particles supported by three different materials (Rh, Au, and ZrO_2_) in H_2_ oxidation has been studied in situ by correlative photoemission electron microscopy (PEEM) and scanning photoemission electron microscopy (SPEM). Kinetic transitions between the inactive and active steady states were monitored, and self-sustaining oscillations on supported Rh particles were observed. Catalytic performance differed depending on the support and Rh particle size. Oscillations varied from particle size-independent (Rh/Rh) via size-dependent (Rh/ZrO_2_) to fully inhibited (Rh/Au). For Rh/Au, the formation of a surface alloy induced such effects, whereas for Rh/ZrO_2_, the formation of substoichiometric Zr oxides on the Rh surface, enhanced oxygen bonding, Rh-oxidation, and hydrogen spillover onto the ZrO_2_ support were held responsible. The experimental observations were complemented by micro-kinetic simulations, based on variations of hydrogen adsorption and oxygen binding. The results demonstrate how correlative in situ surface microscopy enables linking of the local structure, composition, and catalytic performance.

## Introduction

Size and support effects of catalytically active metal particles have been among the most studied phenomena in heterogeneous catalysis for over 50 years.^1−4^ A vast number of ex situ and in situ studies have been carried out by spectroscopic, diffractive, or imaging techniques, both on technological as well as on model catalytic systems.^5−7^ Low-coordinated step/edge sites and interfaces were often found most active.^8−14^ In the present work, we study the effect of various metal/support interfaces on catalytic H_2_ oxidation on Rh by in situ correlative microscopy, i.e., the same area of the same sample is imaged under identical reaction conditions by different microscopies, providing real-time complementary information. Accordingly, interface phenomena and particle size effects can be directly observed by spatially resolved evaluation of surface structure, surface composition, and catalytic performance.

The correlative microscopy concept stems from biological research, where the same cell/tissue structures were studied by light and electron microscopy already in the 1970s.^15,16^ This has been extended to other imaging techniques such as atomic force microscopy, single molecule fluorescence, X-ray tomography, or scanning electron microscopy^17,18^ and has made a huge impact in materials research.^19,20^ In catalysis, correlative microscopy has also been applied, e.g., by combining transmission electron microscopy (TEM) with fluorescence microscopy, atom probe tomography (APT) with scanning transmission X-ray microscopy, scanning photoelectron microscopy (SPEM) with APT, or optical microscopy with confocal and X-ray fluorescence microscopy.^21−25^

On planar Rh samples, catalytic H_2_ oxidation has been studied in a correlative way by various combinations of UV or X-ray photoemission electron microscopy [(UV or X)PEEM], SPEM, or low-energy electron microscopy.^26−28^ Since the initial studies in the 1960s, these techniques have been developed into powerful surface science tools.^29−32^ A wide range of surface processes were investigated, e.g., layer growth,^33^ diffusion,^34^ phase transitions,^35^ adsorption,^36^ oxidation,^37^ or chemical reactions,^38^ with Gerhard Ertls’s work on the oscillating CO oxidation reaction on Pt^39^ being the prime example. XPEEM alone can already provide correlative spectroscopic data and microscopy images, but its capabilities are limited in terms of spectroscopy of sub-micrometer areas and imaging areas larger than several micrometers.^28,40^ Thus, a combination with other techniques is often beneficial. Apart from the known steady states of high and low activity and bistability,^41−44^ previous studies shed light on the spatio-temporal phenomena in catalytic H_2_ oxidation on Rh, including mono- and multi-frequential self-sustaining oscillations,^12,45,46^ coexisting multi-states,^47^ or the emergence of chaos.^48^ For supported Rh particles, such an approach has not yet been applied. Based on the thorough understanding of the mechanism of hydrogen oxidation, it can be used as a test reaction to probe catalytically inactive and active states. By assessing the parameter regions for these states, interface and size effects in different catalysts can be benchmarked.

Catalytic H_2_ oxidation is also relevant in various areas of technology: On one hand, excess electric energy can be stored in the chemical bonds of H_2_ and released on demand in a fuel cell, being one route toward renewable energy generation and storage.^49^ On the other hand, for some applications, traces of H_2_ must be safely removed. This includes, e.g., treatment of fuel cell off-gas,^50^ nuclear power plants,^51^ or feeds of catalytic reactors.^52^ Furthermore, catalytic H_2_ oxidation also occurs in hydrogen gas sensors.^53^

Here, we present the first correlative microscopy study of catalytic H_2_ oxidation on Rh powder aggregates (size of ∼5–30 μm) supported by different materials: A single sample comprising Rh aggregates supported either by Rh, Au, or ZrO_2_ was studied in situ by combining PEEM (providing local reaction kinetics) and SPEM (providing local chemical information). The Rh/Rh system represents a reference for unmodified (quasi-unsupported) Rh, with the particles nonetheless having the same curved/stepped surface morphology as in the Au and Zr-oxide supported systems. Rh/Au was initially prepared to provide an inert support but finally served as a model system for active particles modified by an inactive second metal, possibly by active site blocking^54^ or surface alloying. This configuration is comparable to, e.g., Au- or Cu-modified Ni-based steam reforming catalysts^55,56^ or Au-modified Cu-based catalysts for water gas shift.^57^ As an example for oxide supported metals, the Rh/ZrO_2_ system was used, which is catalytically active in steam reforming,^58^ CO and CO_2_ hydrogenation,^59^ or H_2_ oxidation.^44,60^ Late transition metals (including platinum group metals) supported on various oxides (including ZrO_2_) are well-known for strong metal–support interaction (SMSI),^61−69^ i.e., fully or partially oxide decorated metal surfaces. In the present study, we demonstrate that the mechanisms of surface modification are very different, affecting both steady state and oscillatory hydrogen oxidation, but the modification of steps was crucial in both cases.

**Figure 1:**
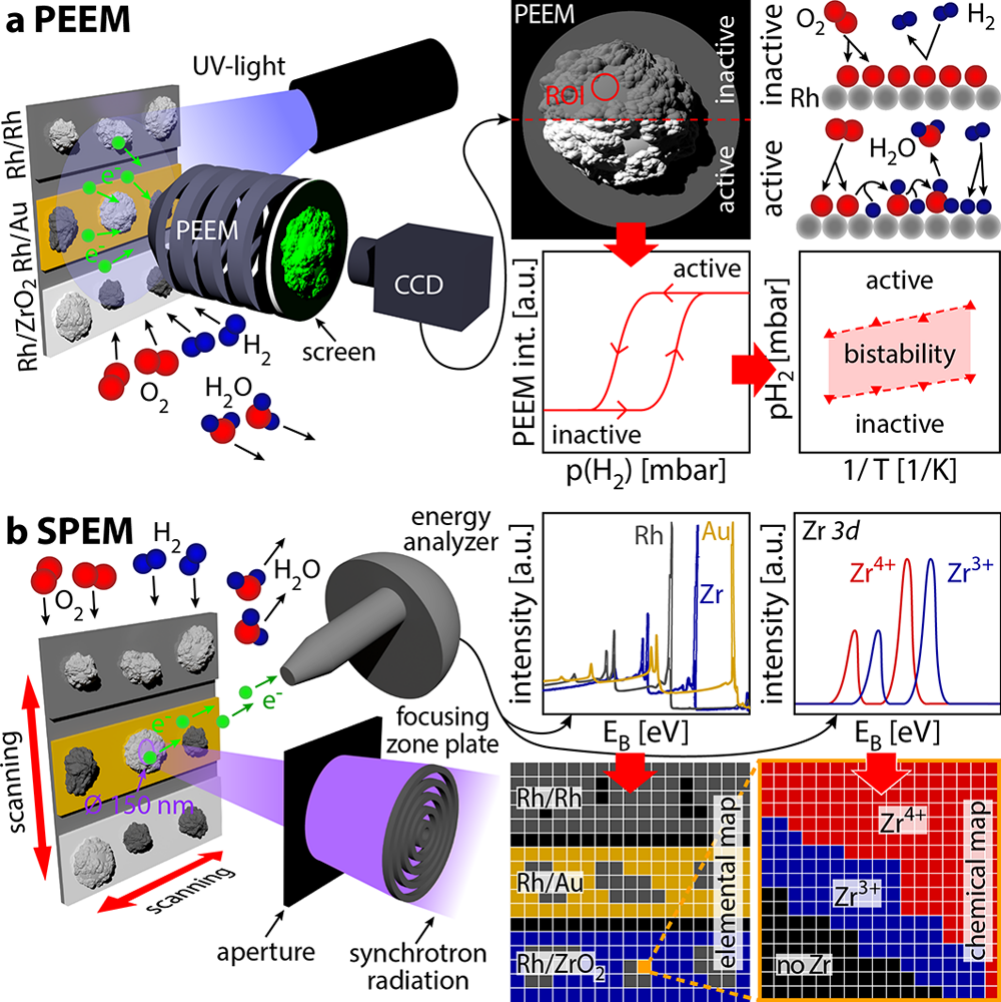
The correlative microscopy approach. (a) In PEEM, the sample is illuminated by UV-light, and the photoemitted electrons form an image on a fluorescent screen. The image brightness is related to the states of catalytic activity shown in the schematic ball models. From the PEEM video sequences, hysteresis curves and kinetic phase diagrams can be obtained for selected regions (ROIs). (b) In SPEM, the sample is raster-scanned under a sub-μm synchrotron X-ray beam, and energy analysis of the emitted photoelectrons provides local X-ray photoelectron spectra (XPS) and elemental/chemical maps on various length scales.

The experiments are illustrated in Figure 1: H_2_ oxidation occurring simultaneously on the three sample regions (Rh/Rh, Rh/Au, Rh/ZrO_2_) was visualized in situ by PEEM. The sample is illuminated by UV-light and the emitted photoelectrons form an image on a fluorescent screen. The image brightness depends on the adsorbate coverage via the work function and is thus directly related to catalytic activity (kinetics by imaging).^70^ Therefore, spatially resolved reaction kinetics, e.g., hysteresis curves and kinetic phase diagrams, can be extracted by analyzing PEEM video sequences.

To complement local kinetics with spatially resolved chemical information, the same sample regions were studied in situ by SPEM under identical reaction conditions, with the sample raster-scanned under a focused sub-micrometer synchrotron X-ray photon probe. The energy distribution of the emitted photoelectrons is measured, resulting in high-resolution X-ray photoelectron spectra (XPS) and elemental/chemical maps on a micrometer scale.

## Results and Discussion

### Kinetic PEEM Studies

PEEM studies were carried out in an ultrahigh vacuum (UHV) setup operated as a flow reactor (for details, see the Supporting Information). First, the kinetic transitions between the steady states of catalytic activity in H_2_ oxidation were studied on Rh/Rh. The reaction follows the Langmuir–Hinshelwood mechanism,^41,71^ i.e., both reactants adsorb on the catalyst before reacting. At low *p*(H_2_)/*p*(O_2_), the system is in an inactive steady state (adsorbed oxygen blocks the hydrogen adsorption, ball model in Figure 1a). Upon increasing *p*(H_2_)/*p*(O_2_), the system switches to a catalytically active steady state. This, and the reverse, occur via kinetic transitions, which can be visualized due to the dependence of the PEEM image intensity on surface coverage: The catalytically inactive oxygen-covered Rh surface has a higher work function than adsorbate-free Rh, resulting in darker PEEM contrast. In turn, the catalytically active surface with low adsorbate coverage appears much brighter. These image contrast variations allow extracting local kinetic information from in situ recorded PEEM videos (kinetics by imaging).^43,45,70^

**Figure 2:**
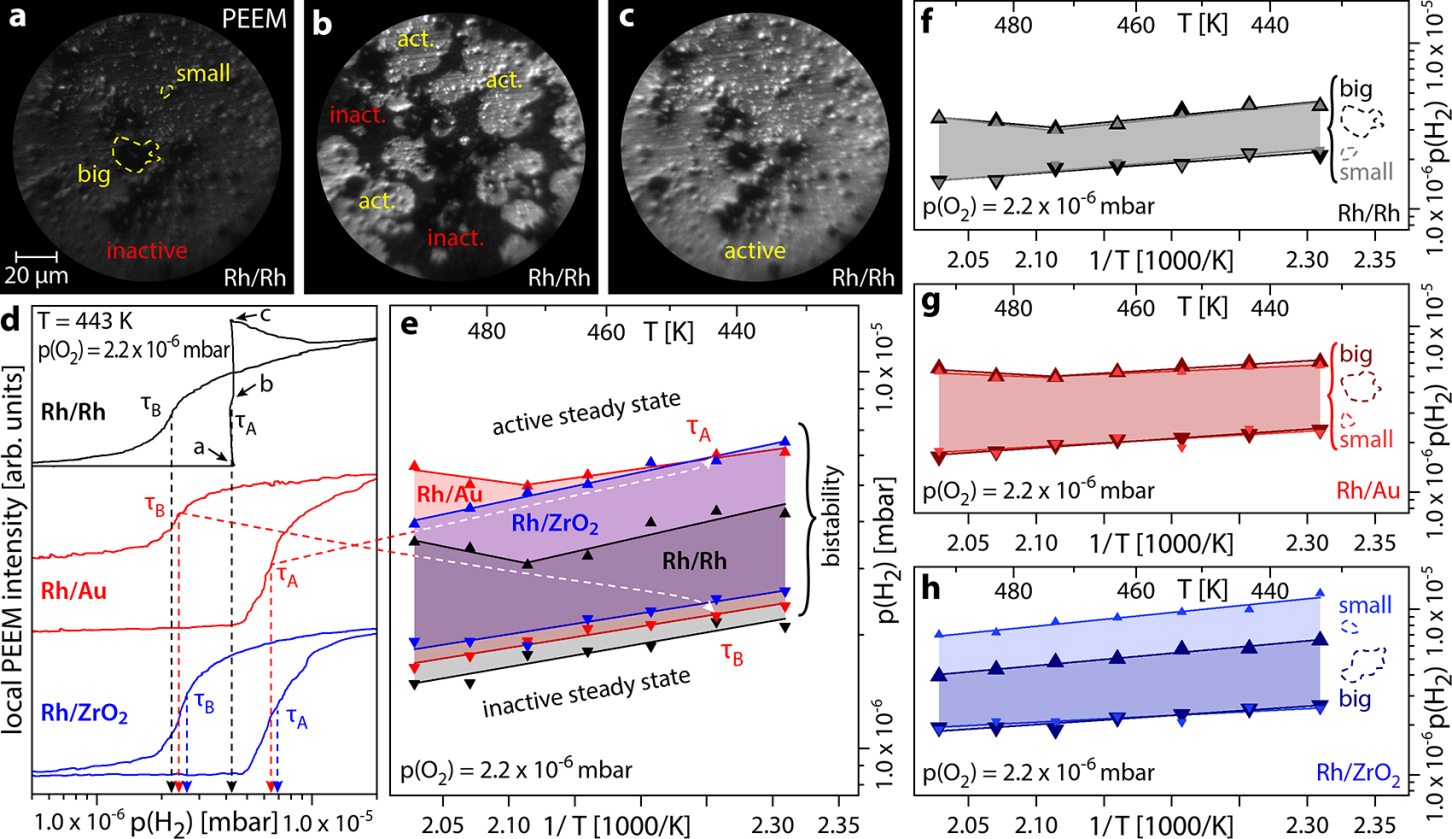
Support and particle size effects in H_2_ oxidation on supported Rh particles. (a) PEEM image of oxygen-covered catalytically inactive Rh particles on Rh support. (b) In situ PEEM image of an ongoing kinetic transition to the catalytically active state at *T* = 443 K, *p*(O_2_) = 2.2 × 10^–6^ mbar, and *p*(H_2_) = 4.2 × 10^–6^ mbar. Dark areas correspond to a catalytically inactive surface, while the bright areas spreading from the Rh particle boundaries indicate a catalytically active Rh surface. (c) Catalytically active surface resulting from the kinetic transition depicted in (b). (d) Hysteresis curves registered during cyclewise variation of p(H_2_) from 5.0 × 10^–7^ mbar to 2.0 × 10^–5^ mbar at constant *T* = 443 K and *p*(O_2_) = 2.2 × 10^–6^ mbar, obtained by processing the local PEEM intensity of a selected ROI on similarly sized Rh particles on Rh foil [black trace, ROI marked in (a)], Au (red trace) and ZrO_2_ (blue trace) support. Conditions of frames (a–c) are marked by arrows. The dashed lines illustrate the construction of a kinetic phase diagram. (e) Kinetic phase diagram for H_2_ oxidation in the temperature range from 433 to 493 K at constant *p*(O_2_) = 2.2 × 10^–6^ mbar for similarly sized Rh particles supported by Rh (black), Au (red) and ZrO_2_ (blue). (f) Same as in (e) but for two differently sized Rh particles (big, black diagram; small, grey diagram) on Rh support. (g) Same as in (f) but for two differently sized Rh particles on Au support. (h) Same as in (f) but for two differently sized Rh particles on ZrO_2_ support.

The observed kinetic transitions are illustrated in Figure 2: Experiments always started from the catalytically inactive state (shown for Rh particles on Rh foil in Figure 2a). Upon increasing *p*(H_2_) at constant *p*(O_2_) and *T*, a kinetic transition to the catalytically active state takes place at a certain *p*(H_2_) value τ_A_. On Rh/Rh this is accompanied by *H*_ads_ fronts nucleating at the perimeter of the particles^44^ and spreading over the whole field of view (Figure 2b). After the kinetic transition, the system stays in the active steady state (Figure 2c). Upon decreasing *p*(H_2_), a reverse kinetic transition to the inactive state will take place at a certain *p*(H_2_) value τ_B_ (Figure 2d), which differs from τ_A_, exhibiting a hysteresis and indicating bistability.^41−44^ From recorded video sequences, the local PEEM intensity can be read out for any region of interest (ROI). For Rh/Rh, a ROI was placed, e.g., on the “big” particle in the middle (marked in Figure 2a) and the local PEEM intensity vs p(H_2_) upon cyclewise variation of *p*(H_2_) is shown as black trace in Figure 2d. The curve exhibits the expected pronounced hysteresis between the kinetic transition points τ_A_ and τ_B_. To examine the effect of different supports, identical experiments were performed for Rh/Au and Rh/ZrO_2_. Similarly sized Rh particles (diameter ∼20 μm) were selected for analysis, and the results for Rh/Au (red) and Rh/ZrO_2_ (blue) are shown in Figure 2d. A hysteresis was also observed for both but with a much wider and shifted loop.

By conducting experiments at different temperatures and plotting the measured τ_A_ and τ_B_ values vs the inverse temperature, kinetic phase diagrams can be constructed. For Rh/Rh (Figure 2e, black), the unexpected sharp bend in the τ_A_ line can be attributed to oxygen-induced surface Rh restructuring and corresponding roughness changes at a certain temperature, reported for several single crystals,^72−75^ Rh nanoparticles,^62,76^ or needle-shaped specimens.^77^ Due to the rounded shape of the Rh particles/aggregates, exposing plenty of differently oriented stepped surfaces (Figure S5), similar behavior can be expected in the present case.

The kinetic phase diagram of Rh/Au (red) is similar to the one of Rh/Rh, but τ_A_ and τ_B_ are both shifted to higher p(H_2_). Since (bulk) Au is catalytically inactive in H_2_ oxidation due to the lack of dissociative adsorption of the reactants,^78,79^ and no interaction between the Rh particles and the Au support is expected, this seems unusual and will be explained below.

For Rh/ZrO_2_ (blue diagram), the τ_A_ and τ_B_ values are also shifted to higher p(H_2_) in comparison to Rh/Rh, but the bistability area does not exhibit a sharp bend in τ_A_. The shift toward higher *p*(H_2_) has been discussed in our previous work.^44^ Due to the electron density jump across the metal/oxide interface, the binding energy for oxygen is modified in close vicinity of the interface. Since the energetics govern the adsorption kinetics, the local oxygen/hydrogen adsorption equilibrium is shifted toward oxygen, necessitating compensation by higher p(H_2_) to induce kinetic transitions. Furthermore, it appears that the presence of the metal/oxide interface inhibits the oxygen-induced restructuring observed on Rh/Rh and Rh/Au.

Besides the “big” Rh particles, other differently sized particles are present in the field of view (Figure 2a–c) and the in situ recorded video sequences contain kinetic information for all particles at inherently identical conditions, allowing the addressing of size effects. Several particles of various sizes were analyzed, and representative examples were selected. Figure 2f,g shows the kinetic phase diagrams analogous to Figure 2e, but additionally for a set of “small” particles (∼5 μm). The corresponding PEEM images are shown in Figure S1. Comparison of diagrams for differently sized particles reveals interesting features: For Rh/Rh, the kinetic phase diagrams are identical within experimental accuracy, which is expected as there are no interface effects between the Rh particles and Rh support. The same seems true for Rh/Au, but the shift in comparison to Rh/Rh remains to be explored below. In contrast, for Rh/ZrO_2_, there is a clear particle size effect: The τ_A_ values of the “small” particle are shifted toward higher *p*(H_2_), while the τ_B_ values remain basically identical. This demonstrates the contribution of the particle/support interface to the catalytic activity. For smaller particles, the ratio of perimeter/interface length to surface area is higher than that for bigger particles: Smaller particles are more strongly influenced by the interface than bigger ones.

The PEEM results unambiguously show interface and particle size effects but lack detailed chemical information. In particular, the behavior of the Rh/Au system cannot be explained by PEEM alone, calling for a chemically sensitive technique.

### Chemically Resolved SPEM Studies

Therefore, SPEM experiments, with the SPEM chamber also operating as a flow reactor, were performed using a photon energy of 720 eV at the ESCA Microscopy beamline (Elettra synchrotron), as described in detail in the Supporting Information along with information on the spectra deconvolution procedures.

**Figure 3:**
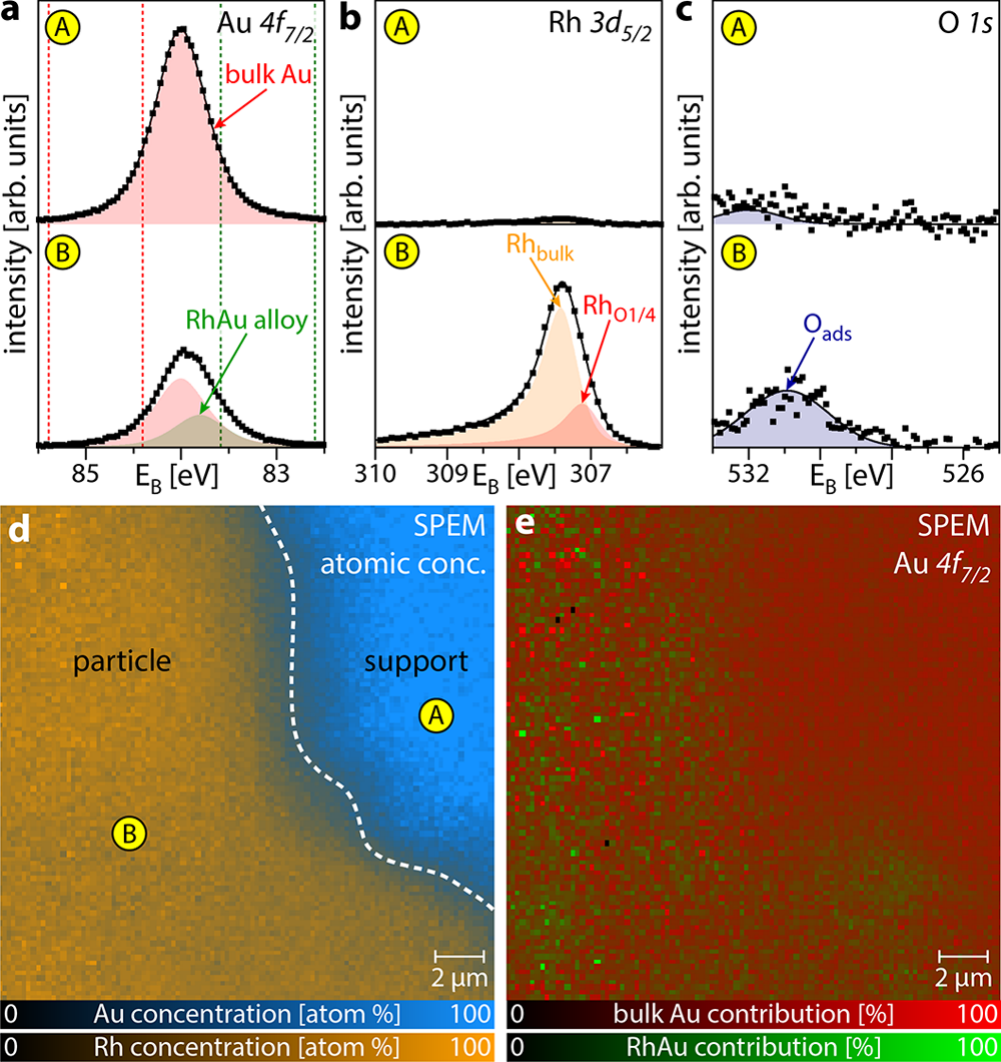
Surface alloying on a Au-supported Rh particle in the catalytically active state at *T* = 453 K, *p*(O_2_) = 2.2 × 10^–6^ mbar, and *p*(H_2_) = 4.0 × 10^–6^ mbar. (a) Local Au 4f_7/2_ XPS spectra at spots A and B indicated in (d). The red component is related to bulk Au, while the green component is related to the formation of a RhAu surface alloy. (b) Local Rh 3d_5/2_ XPS spectra at the spots A and B indicated in (d). The orange component corresponds to bulk Rh, while the red component is related to Rh bound to adsorbed oxygen. (c) Local O 1s XPS spectra at the points A and B indicated in (d). The single blue component corresponds to adsorbed oxygen. (d) SPEM chemical map of the atomic concentrations of Au (blue) and Rh (orange) at the boundary of a Rh particle. The particle boundary is indicated by a white dashed line. (e) Au 4f SPEM chemical map of the distribution of the two different Au species. The field of view is identical to (e). The energy windows used for constructing the map are indicated by dashed red and green lines in (c) and given in Table S2.

In Figure 3, the same “big” Rh particle on Au as in PEEM was probed in the catalytically active state (the Rh/Rh system provides no chemical contrast). Figure 3a shows high-resolution in situ Au 4f_7/2_ spectra of the two spots marked in Figure 3d: Spot A on the support and spot B on the Rh particle. While spot A shows expected bulk Au, significant Au amounts are detected on the Rh particle as well. There are three possible reasons: First, the cleaning procedure includes Ar^+^-ion sputtering, possibly leading to deposition of support material onto the Rh particles. Such redeposition has been observed on several substrates and under various sputter conditions.^80,81^ Second, Au adatoms can diffuse on platinum group metals at temperatures as low as 300 K.^82,83^ Third, kinetic transitions in a surface reaction may cause a large-scale redistribution of adsorbed Au.^84^ Furthermore, the spectra show two distinct Au species, bulk Au (support material, red) and another one only on the Rh particle (green). We attribute this second component to the formation of a RhAu surface alloy, in analogy to studies of thin Au films on Al, Rh, or Ru.^85−87^

The corresponding Rh 3d_5/2_ spectra (Figure 3b) indicate no presence of Rh on the support material. We conclude that the presence of Au on the Rh particles is caused by a combination of the effects mentioned above: Au atoms are deposited on the Rh particle in close vicinity to the interface due to sputtering, from where they diffuse and migrate, finally covering the whole particle. The spectrum on the particle, deconvoluted following a previously established procedure,^47^ consists of a bulk Rh component (Rh_bulk_, orange) and a component related to oxygen-bound Rh (Rh_O1/4_, red), which unambiguously indicates the catalytically active state.^47^ The O 1s spectra (Figure 3c) highlight that the Au support does not take part in the reaction (no oxygen is present in spot A), while the spectrum for the Rh particle (spot B) agrees with our previous observations.^47^

Assuming a simple homogeneous layer model for analysis of the Au 4f_7/2_ versus Rh 3d_5/2_ absolute intensities yields an average Au thickness of below one monolayer. Au is, however, known to form islands and multi-layered clusters^84,87^ on Rh, resulting in a significantly more complex morphology.

Using the Au 4f_7/2_ and Rh 3d_5/2_ signals after correction for the different inelastic mean free paths and X-ray cross-sections, a chemical map of the atomic concentrations of Au (blue) and Rh (orange) was constructed (Figure 3d). The map reveals that there is hardly any gradient in the Au concentration on the Rh particle with the entire particle being covered. A Au 4f_7/2_ chemical map detailing the lateral distribution of the two different Au species is displayed in Figure 3e, whereby the relative contributions of the individual species to the total Au 4f_7/2_ signal are shown. The map completes the picture and supports our component assignment of RhAu on the particle. The individual contributions to the maps displayed in Figure 3d,e are depicted in Figure S2.

Based on SPEM, the unexpected behavior of Rh/Au in PEEM can be explained: Au atoms migrating onto the Rh particle are very likely located at the Rh step edges, in line with previous observations on Pt, Rh, or Ru.^54,83,87^ Since the Rh step sites are crucial for dissociative hydrogen adsorption^88^ and thus for switching to the active state, their lower activity due to RhAu alloy formation must be compensated by a higher *p*(H_2_), shifting the kinetic phase diagram as observed.

**Figure 4:**
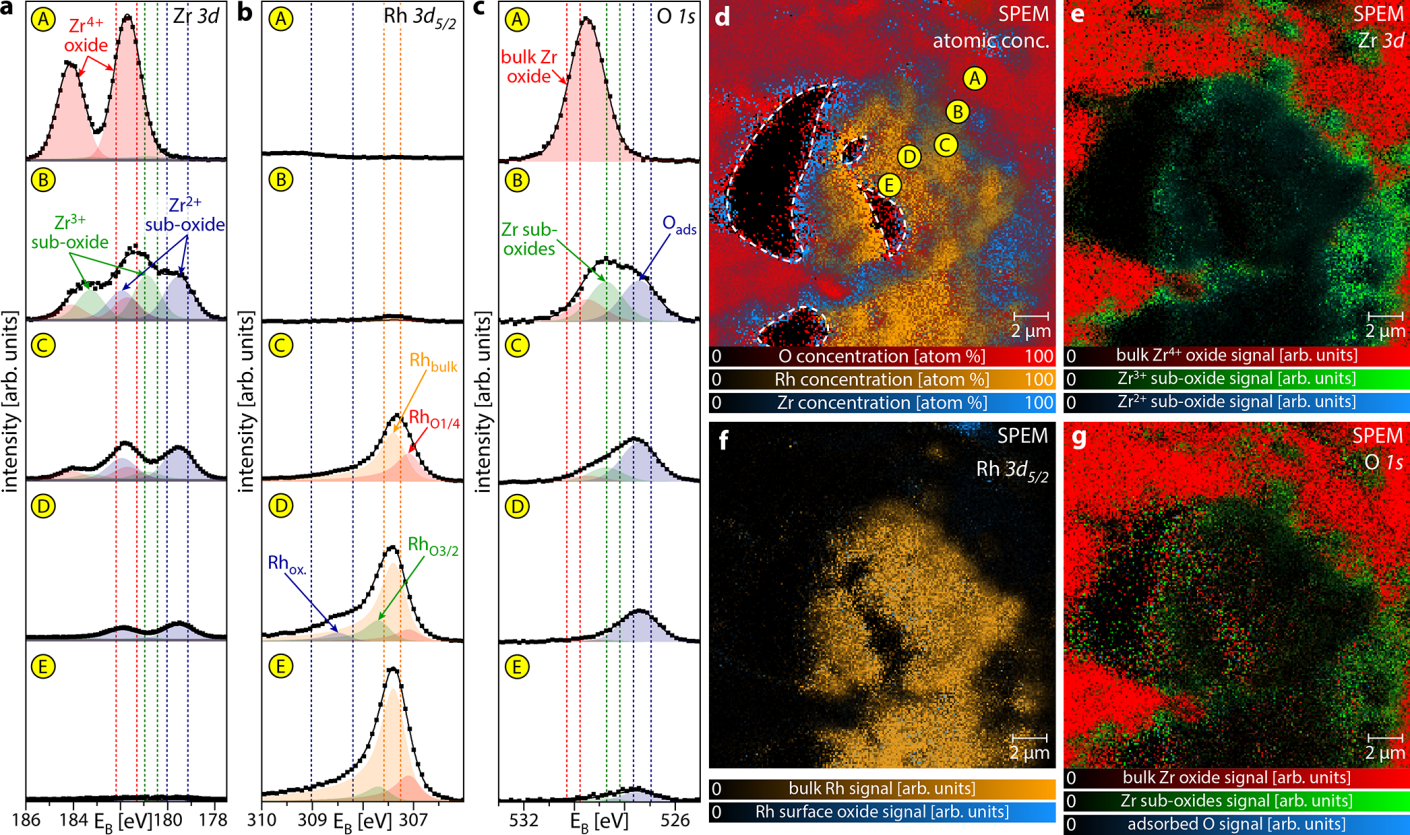
Metal/oxide interface effects on ZrO_2_ supported Rh particles in the catalytically active state at *T* = 453 K, *p*(O_2_) = 2.2 × 10^–6^ mbar, and *p*(H_2_) = 4.0 × 10^–6^ mbar. (a) Local Zr 3d XPS spectra for spots A to E indicated in (d), revealing several oxide components (Zr^4+^ bulk oxide–red; Zr^3+^ sub-oxide–green; Zr^2+^ sub-oxide–blue). (b) Local Rh 3d_5/2_ XPS spectra for spots A to E indicated in (d). In addition to bulk Rh (orange) and Rh surface oxide (blue), two components related to differently oxygen-bound Rh are present (green and red). (c) Local O 1s XP spectra for spots A to E indicated in (d), showing different oxygen species (bulk Zr oxide–red; Zr sub-oxides–green; adsorbed oxygen–blue). (d) SPEM chemical map of the atomic concentrations of O (red), Rh (orange), and Zr (blue). The white dashed lines indicate areas not imaged due to the setup geometry. (e) Zr 3d SPEM chemical map for different Zr-oxide species. The field of view is identical to (d). The spectra components correspond to the species in (a), where the dashed lines indicate the energy windows used for constructing the map. (f) Rh 3d SPEM chemical map for different Rh species (bulk Rh–orange; Rh surface oxide–blue). The field of view is identical to (d). The energy windows used for constructing the map are indicated by dashed lines in (b). (g) O 1s SPEM chemical map for different oxygen species. The field of view is identical to (d). The spectra components correspond to the species in (c), where the dashed lines indicate the energy windows used for constructing the map. All energy windows for constructing the chemical maps are also given in Table S2.

In analogy to Rh/Au, in situ SPEM of the “big” Rh particle on ZrO_2_ in the active state is shown in Figure 4. In Figure 4a–c, high-resolution Zr 3d, Rh 3d_5/2_, and O 1s XPS spectra are displayed for spots A to E placed along a line from the oxide support to the Rh particle center. The Zr 3d spectra (Figure 4a) display three different Zr^*x*+^ species (Zr^4+^ bulk-oxide, red; Zr^3+^ sub-oxide, green; and Zr^2+^ sub-oxide, blue), identified based on previous XPS studies of thin ZrO_*x*_ islands/films on Rh(111)^61^ or Pt(111)^68^ and of the initial oxidation of Zr.^89,90^ Similarly, detailed analysis of the Rh 3d_5/2_ spectra (Figure 4b) reveals the presence of bulk Rh (Rh_bulk_, orange), two components associated with oxygen-bound Rh (Rh_O1/4_, red, and Rh_O2/3_, green) as well as a component related to Rh surface oxide (Rh_O*x*_, blue), in accordance with our previous work.^27,47^ The O 1s spectra (Figure 4c) complete the picture: One component is associated with bulk ZrO_2_ (red), one component is related to the Zr sub-oxides (green), and one component represents oxygen adsorbed on Rh (blue).

Combining all of the information yields the following: At spot A (i.e., on the support, several μm away from the particle), there is bulk-like ZrO_2_. At spot B, still on the support, but near the metal/oxide interface, Zr sub-oxides are present in addition, but there are no significant amounts of Rh. This can be understood by partial reduction of ZrO_2_, possibly by hydrogen spillover, as, e.g., observed for Rh or Pt particles on ZrO_2_.^61,91,92^ Computational studies have shown that reduction of nano-sized ZrO_2_ is facilitated in comparison to bulk ZrO_2_, making partial reduction feasible under the present conditions.^93^ On the Rh particle near the metal/oxide interface (spot C), significant amounts of zirconia are present mostly as Zr sub-oxides, while the RhO_1/4_ component again indicates the active state. Further toward the particle center (spot D), the amount of Zr sub-oxides decreases and the Rh 3d_5/2_ spectrum reveals an unexpected shoulder due to a distinct Rh surface oxide. It seems that small amounts of Zr sub-oxides locally enhance the oxidation of Rh, probably as a result of the stronger oxygen bonding close to the metal/oxide interface.^44,94^ Even more toward the particle center (spot E), there is no Zr present and the Rh surface is in the active state.

Based on reports that the growth of ZrO_*x*_ 2D-islands preferentially started at step and defect sites of a Pt substrate,^95,96^ it can be suggested that the ZrO_*x*_ layers on Rh near the interface may originate from a limited mobility of Zr–O species at temperatures around 450 K. However, the observed formation of small ZrO_*x*_ islands closer to the Rh center rather takes place by sputter-induced support deposition on the Rh particles, where it forms small, hardly mobile oxide islands.

A chemical map (Figure 4d) of the atomic concentrations of O (red), Rh (orange), and Zr (blue) was constructed from the O 1s, Rh 3d_5/2_, and Zr 3d signals. The map shows a significant coverage of ZrO_*x*_ on the Rh particle. The island density of ZrO_*x*_ is, however, not as uniform as for Au on Rh, but decreases toward the particle center, which remains free of ZrO_*x*_. In addition, the Zr/O atomic ratio differs up to 50% between the support (appearing purple) and near the interface (appearing blue). This is also reflected in the Zr 3d chemical map (Figure 4f), constructed using the same three components as in the spectra (Figure 4a), which shows the absolute background-normalized contributions of the individual species to the total Zr 3d signal. The Rh 3d_5/2_ chemical map (Figure 4f), constructed using the absolute, background-normalized contributions of the individual species to the total Rh 3d_5/2_ signal reveals mainly bulk metallic Rh (orange) being present on the “big” particle, while small patches of Rh surface oxide (blueish dots) are also formed. Smaller particles (e.g., in the top right corner) appear to be strongly oxidized. The corresponding O 1s chemical map (Figure 4g), again displaying the absolute background-normalized contributions of the individual species to the total O 1s signal, shows the expected gradient between support (appearing mainly red), interface region (appearing green), and the center region (appearing in a slight bluish tint). The individual contributions to the maps displayed in Figure 4d–g are depicted in Figure S3.

The SPEM results demonstrate an unexpected complexity of the seemingly simple metal/support interfaces: Due to the mobility of Au atoms, the Rh particles were decorated by significant amounts of Au, strongly modifying the catalytic properties by RhAu alloy formation. In the Rh/ZrO_2_ system, due to the formation of ZrO_*x*_ islands, a complex interface region spanning several micrometers has been formed, resembling strong metal–support interaction (SMSI) states observed on several platinum group metal nanoparticles on various oxides.^61−64,66,68,69,97^ Furthermore, the previously identified modification of binding energies at the metal/oxide interface^44,94^ appears to be complemented by the formation of sub-stoichiometric Zr oxides, hydrogen spillover onto the ZrO_*x*_ support near the particle, and a narrow region where Rh surface oxide is present, altogether resulting in the observed support-induced modifications of the catalytic properties (Figure 2).

### Oscillating Reaction Mode

Catalytic H_2_ oxidation may exhibit self-sustaining oscillations between the active and inactive state at particular constant reaction parameters, as observed on polycrystalline Rh foils^45−47^ and hemispherical apexes of nanometer- and micrometer-sized Rh specimens.^12,13,98^ The oscillation frequency and the parameter window for oscillations are very sensitive to surface structure and composition,^12,45−47^ turning the oscillating H_2_ oxidation on Rh into a sensitive probe. The well-established mechanism of oscillations (Figure S4) is based on the formation and depletion of subsurface oxygen.^45−47^ However, as for supported Rh particles, self-sustained oscillations in H_2_ oxidation have so far not been observed, their existence and parameter space should be explored.

**Figure 5:**
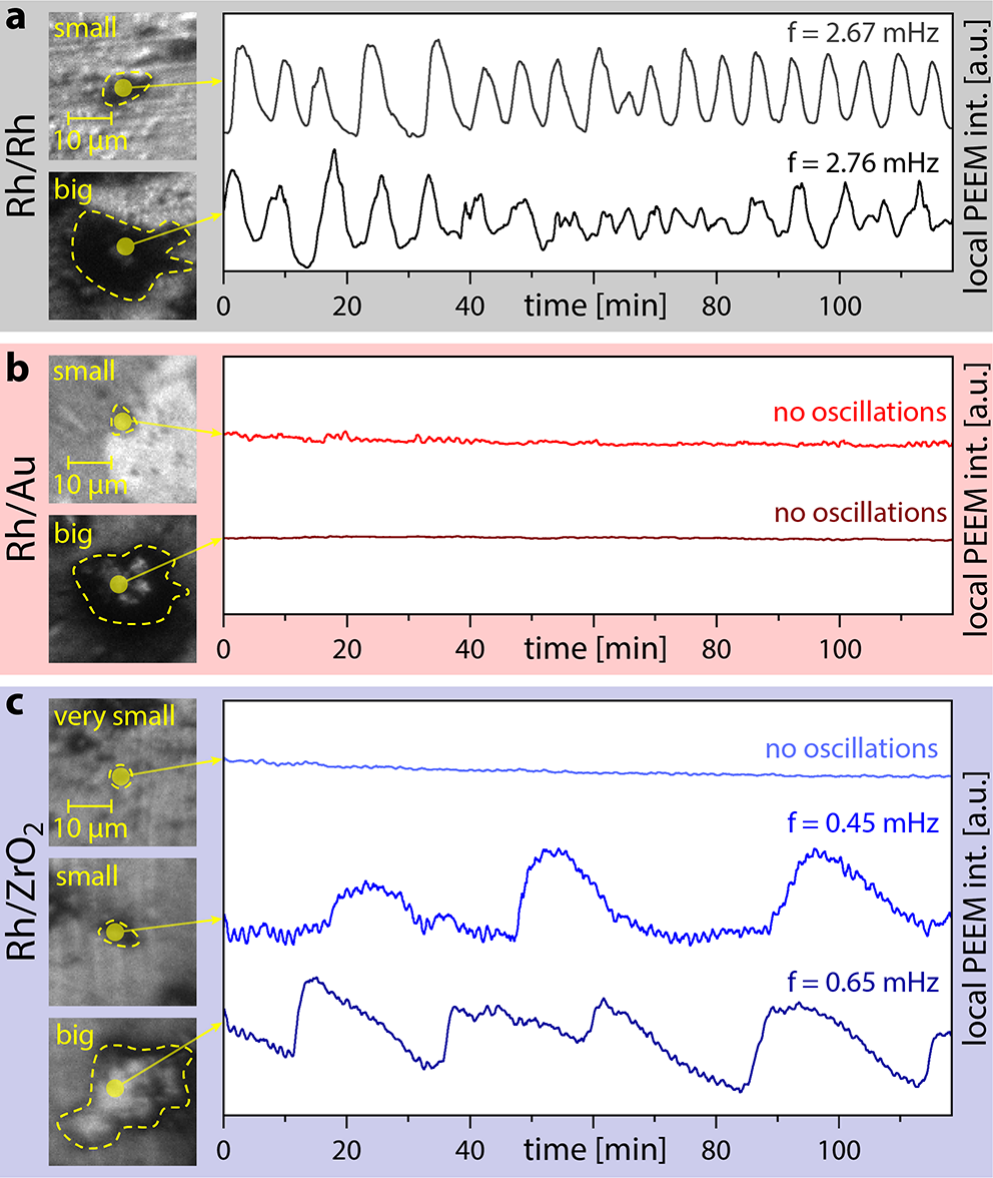
Visualizing support and particle size effects by the oscillating mode of catalytic H_2_ oxidation on Rh particles at *T* = 453 K, *p*(O_2_) = 2.2 × 10^–6^ mbar, and *p*(H_2_) = 1.7 × 10^–6^ mbar. (a) PEEM image cutouts (left) and local PEEM intensity time series (right) for differently sized Rh particles supported on Rh. The ROIs for constructing the time series are indicated in the cutouts. (b) Same as in (a) but for Rh particles on Au support. (c) Same as in (a) but for Rh particles on ZrO_2_ support.

At constant external parameters, oscillations were visualized by PEEM for Rh/Rh and Rh/ZrO_2_ (but did not occur for Rh/Au). For Rh/Rh, selected PEEM image cutouts for the “big” and “small” particles already studied in the steady states and the corresponding local PEEM intensity time series are shown in Figure 5a. Both particles exhibit the same oscillation frequency within the experimental accuracy. It is important to note that while the Rh substrate acts as excitable medium and therefore also exhibits oscillations, the nucleation of the reaction fronts transmitting the oscillations always takes place on the Rh particle perimeters acting as pacemakers.^13,94^

For Rh/Au, PEEM images and local intensity curves are displayed in Figure 5b. No oscillations occur, with the particles remaining in the inactive state. This can be explained in light of SPEM experiments and the mechanism of oscillations: Au atoms migrated onto the Rh particle are located preferentially at/in steps, hindering the formation of subsurface oxygen and thus the switch to the active state.

In Figure 5c, the corresponding results for Rh/ZrO_2_ are shown. In addition to the “big” and “small” particles, even “very small” ones were studied, for which no oscillations were observed (exemplary curve in Figure 5c), apparently due to their oxidized state resulting from ZrO_*x*_-promoted Rh-oxidation. Oscillations observed for “small” and “big” Rh particles exhibited significantly lower frequency than those observed for Rh/Rh, which depended on particle size. We attribute this to the Rh steps being partly blocked by ZrO_*x*_ and the resulting stronger oxygen bonding. Accordingly, the oscillation cycle slows down. Differently sized Rh particles are differently affected and thus exhibit different oscillation frequencies, due to their different ratios of ZrO_*x*_-modified (interfacial) vs -unmodified Rh surface areas.

In order to assess the curved/stepped particle shape and structure, post-reaction TEM analysis (Figure S5) was performed on cross-sections of some of the studied particles. The composition of particle surfaces and metal/support interface regions was characterized by combined scanning-TEM/energy dispersive X-ray fluorescence (EDX), confirming the in situ results.

### Micro-Kinetic Model Simulations

To better understand the experiments, micro-kinetic model simulations of H_2_ oxidation were performed, based on the Langmuir–Hinshelwood mechanism and by adapting a model of McEwen et al.^98,99^

For representing Rh/Rh, parameters similar to those used in our previous work were used.^12,45−47^ Reflecting the PEEM and SPEM results to model Rh/Au, the hydrogen sticking coefficient was adjusted to simulate hindered dissociative hydrogen adsorption and the parameter corresponding to surface “roughness” was decreased, both resulting from RhAu surface alloy formation. To model Rh/ZrO_2_, parameters were modified to simulate the stronger oxygen binding/adsorption^44^ and the changed energetics of subsurface oxygen formation in the metal/oxide interface regions (for details, see the Supporting Information).

**Figure 6:**
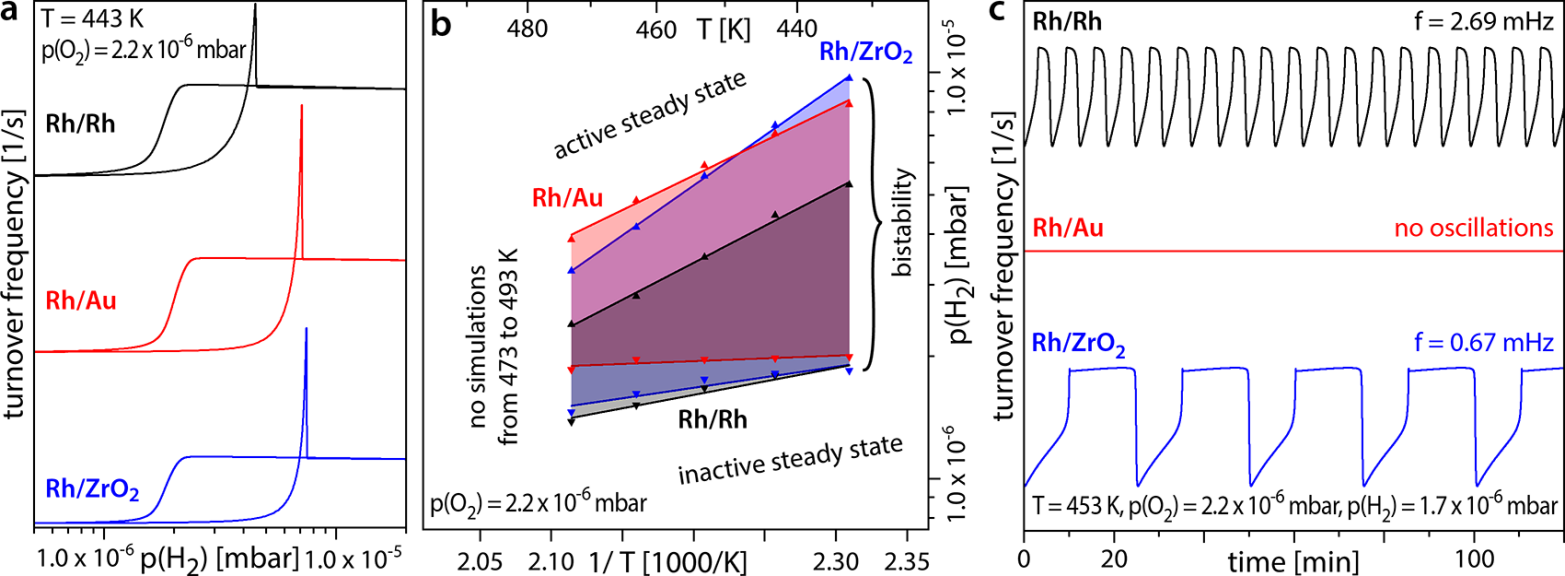
Results of the micro-kinetic model simulations. (a) Simulated turnover frequency curves upon cyclewise variation of *p*(H_2_) from 5.0 × 10^–7^ to 2.0 × 10^–5^ mbar at constant *T* = 443 K and *p*(O_2_) = of 2.2 × 10^–6^ mbar for three model configurations representing Rh/Rh (black), Rh/Au (red), and Rh/ZrO_2_ (blue). (b) Simulated kinetic phase diagram for H_2_ oxidation in the temperature range from 433 to 473 K at constant *p*(O_2_) = 2.2 × 10^–6^ mbar for the same model configurations as in (a). (c) Simulations of the oscillating reaction mode at constant *T* = 453 K, *p*(O_2_) = 2.2 × 10^–6^ mbar, and *p*(H_2_) = 1.7 × 10^–6^ mbar for the same model configurations as in (a).

Figure 6 presents the results of the micro-kinetic model simulations: In Figure 6a, traces of the reaction rate (turnover frequency; TOF) upon cyclewise variation of p(H_2_) are displayed for the three different model configurations, i.e., Rh/Rh (black), Rh/Au (red), and Rh/ZrO_2_ (blue). Simulations were performed for the external parameters of the PEEM experiments, allowing a direct comparison (cf. Figure 2d). By repeating simulations for different temperatures, a kinetic phase diagram can be constructed (Figure 6b), similar to the experimental diagram in Figure 2e. Simulations were performed only for 433–473 K, because oxygen-induced surface restructuring was not included in the model. Simulations of the oscillating mode were also performed for all three configurations. The simulated TOF time series are shown in Figure 6c, corresponding to the big Rh particles in Figure 5.

The calculations/simulations reproduce the experimental behavior well: Realistic variations of hydrogen adsorption and oxygen binding, mimicking the effects of the various Rh/support interfaces, quantitively reproduce the shifts in the kinetic phase diagrams as well as the alteration of the oscillatory behavior (oscillation frequency and inhibition of oscillations).

## Conclusions

In summary, the catalytic behavior of stepped Rh particles supported by three different materials (Rh, Au, and ZrO_2_) has been visualized in situ by PEEM for H_2_ oxidation, a probe reaction that is also relevant in many areas of technology. Kinetic transitions between the catalytically inactive and active steady states were studied and self-sustaining oscillations on supported Rh particles were observed for this reaction for the first time. Supported Rh particles show different catalytic properties in H_2_ oxidation, depending on the support material and particle size. The oscillating reaction mode varies from particle-size-independent (Rh/Rh) via size-dependent (Rh/ZrO_2_) to fully inhibited oscillations (Rh/Au).

To interpret the PEEM results, in situ SPEM studies were performed for the same sample under the same reaction conditions, i.e., in a correlative way. The SPEM results identified the presence of support material on the Rh particles during the reaction to cause their different behavior. For Rh/Au, the formation of a RhAu surface alloy was observed, and for Rh/ZrO_2_, the formation of substoichiometric Zr oxides, hydrogen spillover onto the ZrO_2_ support, and a narrow region of enhanced Rh-oxidation were detected. Combining the real-time PEEM data and chemical information from SPEM, the catalytic properties of Rh particles on different supports can be explained, underpinning the effectiveness of the correlative approach. The experimental observations were complemented by micro-kinetic model simulations of kinetic phase diagrams and oscillating time series for catalytic H_2_ oxidation on various Rh surfaces. Through realistic variations of hydrogen adsorption and oxygen binding, resulting from the observed modification of stepped Rh surfaces, the experimental findings could be confirmed.

A small particle size and support materials library was created by combining various Rh particle sizes and several support materials in a single sample, which was studied by a combination of microscopy techniques able to provide local reaction kinetics and local chemical information. As the mechanism of catalytic H_2_ oxidation on Rh is well understood, this probe reaction can be used to benchmark interface and particle size effects in catalysis, an approach that could be extended to other important catalytic reactions.

## Supporting Information Available

Experimental and computational procedures, PEEM images of the supported Rh particles, SPEM maps of the individual contributions to the chemical maps, details on the oscillation mechanism, TEM/EDX characterization of some of the Rh particles, and energy windows used in constructing the chemical maps (PDF)
