# Supplementary material for: Imaging Interface and Particle Size Effects by In Situ Correlative Microscopy of a Catalytic Reaction
Source: ACS Catal. 2023 May 23;13(11):7650–60. doi: 10.1021/acscatal.3c00060 (PMC10242684; doi:10.1021/acscatal.3c00060)
Supplement: Supplementary file 1 — cs3c00060_si_001.pdf [file cs3c00060_si_001.pdf]

Supporting Information

Imaging interface and particle size effects by *in situ* correlative microscopy of a catalytic reaction

*Philipp Winkler[1], Maximilian Raab[1], Johannes Zeininger[1], Lea M. Rois[1], Yuri Suchorski[1], Michael Stöger-Pollach[2], Matteo Amati[3], Rahul Parmar[3], Luca Gregoratti[3], and Günther Rupprechter[1, *]*

[1] Institute of Materials Chemistry, TU Wien, Getreidemarkt 9, 1060 Vienna, Austria
[2] University Service Center for Transmission Electron Microscopy, TU Wien, Wiedner Hauptstraße 8-10, 1040 Vienna, Austria
[3] Elettra-Sincrotrone Trieste S.C.p.A., SS 14 km 163.5 in AREA Science Park, 34149 Trieste, Italy

[*] E-Mail: guenther.rupprechter@tuwien.ac.at

Experimental procedures

Sample Preparation

The sample was fabricated from a Rh foil (10 x 3 mm, thickness 0.2 mm, MaTeck, 99.9%), an Au foil (10 x 3 mm, thickness 0.2 mm, MaTeck, 99.9%) and an oxidized Zr foil (10 x 3 mm, thickness 0.2 mm, MaTeck, 99.8%), all mounted onto a Ta foil (10 x 12 mm, thickness 0.2 mm, MaTeck, 99.9%) for mechanical stability. The Rh powder aggregates ("Rh black", 5-30 μm size, Alfa Aesar, 99.9%) were suspended in acetone and mechanically pressed on the substrates after solvent evaporation.

The sample was cleaned in UHV by repeated cycles of Ar^+ ion sputtering (p(Ar) = 5.0×10^{-6} mbar, ion energy 1 keV, T = 300 K) and consecutive chemical treatment in oxygen ($p(O_2) = 5 \times 10^{-7}$ mbar, T = 773 K) and hydrogen ($p(H_2) = 5 \times 10^{-6}$ mbar, T = 773 K). Cleanliness of the sample was verified before each experiment by lab-XPS or by SPEM. The sample temperature was measured by a K-type thermocouple spot-welded to its front.

PEEM experiments

The PEEM experiments were carried out in a multipurpose UHV setup consisting of separate chambers for PEEM and XPS, interconnected by a sample transfer tunnel. The PEEM chamber is equipped with sample cleaning facilities, a deuterium discharge UV lamp (Heraeus D200F, photon energy ~6.5 eV), a PEEM (Staib Instruments PEEM 150), a quadrupole mass spectrometer (MKS e-Vision 2) and a high-purity gas supply (Ar, H_2, O_2: 99.999%). In addition to similar sample cleaning and gas dosing-facilities, the XPS chamber is equipped with a twin anode x-ray source (SPECS XR-50) and a hemispherical energy analyzer (SPECS Phoibos 100).

The ongoing H_2 oxidation reaction was monitored by PEEM and the images recorded by a high-speed CCD camera (Hamamatsu C11440-42U30). Calibration of the PEEM magnification was performed by comparison of PEEM images with optical micrographs of the same sample areas. To prove sample cleanliness, XPS spectra were acquired from one circular spot of 500 μm diameter per support material using Mg K_α radiation with the energy analyzer axis oriented perpendicular to the sample surface (take-off angle 90°).

SPEM experiments

The SPEM experiments were performed at the "ESCA Microscopy" beamline of the Elettra synchrotron facility, which has been described in detail elsewhere *(S1)*. Summarizing, the end station consists of three connected UHV sub-chambers: The sample is introduced to the system via a fast-entry load lock attached to the first chamber. Using magnetic transfer arms and wobble sticks, the sample can be moved under UHV to a preparation chamber, which is equipped with facilities for Ar^+ ion sputtering, a high purity gas supply (Ar, H_2, O_2: 99.999%) and an Auger electron spectrometer for checking the sample cleanliness. After preparation, the sample is moved in UHV to the SPEM chamber. A zone plate optical system provides a small focused photon probe (spot diameter 0.13 μm) on the sample surface, while the analyzed surface region is selected by a piezo specimen positioning and scanning system. The emitted photoelectrons are

collected within an 8 eV kinetic energy window by a hemispherical energy analyzer equipped with a 48 channel detector.

The SPEM was operated in two modes: In the microspectroscopy mode, an XPS spectrum was collected from a single microspot on the sample surface, while in the imaging mode, the sample surface was mapped by synchronized-scanning the sample with respect to the photon probe. In the imaging mode, a 48 points XPS spectrum covering the chosen energy window was recorded for each pixel in the image. This allows determination of the spatial distribution of different chemical species *(S2)* and thus the creation of chemical maps and spatial profiles. The overall energy resolution of the system is 0.3 eV *(S3)* and due to the setup geometry, electrons emitted at an angle of 60° from the surface normal are registered. Spectra were taken at a photon energy of 720.2 eV and the energy scale was calibrated against the energy of the Au $4f_{7/2}$ peak with a binding energy of 84.0 eV on the Au part of the sample. The absence of drifts in photon energy and photon flux was verified at regular intervals between the experiments by Au $4f_{7/2}$ spectra on the Au part of the sample.

All spectra in the present work are representative examples of the described states and were deconvoluted using a (partially asymmetric) pseudo-Voigt line shape *(S4)*, as implemented in the EccentricXPS package (Version 1.101) written by Ole Lytken at the Lehrstuhl für Physikalische Chemie II, Universitaet Erlangen-Nuernberg, Germany *(S5)* for Igor Pro (WaveMetrics Inc., Lake Oswego, OR, USA) in combination with a Shirley background *(S6)*. The spectral components are based on literature data (see details below) and were refined by considering a whole ensemble of spectra, including reference spectra of the clean, catalytically active and catalytically inactive surfaces.

The Au $4f_{7/2}$ spectra were deconvoluted using two components: The symmetric bulk Au component was set at a fixed binding energy of 84.0 eV, while FWHM and Gaussian/Lorentzian (G/L) ratio were varied in order to best reproduce the spectrum of the clean Au support. The symmetric RhAu surface alloy component, having the same FWHM and G/L ratio as the bulk Au component, was added in order to convincingly reproduce all other Au $4f_{7/2}$ spectra. The chemical shift of the RhAu component was adjusted to best reproduce these spectra, finally equaling -0.2 eV, in line with previous literature data on the Rh/Au system *(S7)*.

The deconvolution of the Rh $3d_{5/2}$ spectra followed our previously established procedure *(S8, S9)*: The spectra include up to six components, which are required in order to convincingly reproduce all spectra of clean, catalytically active, catalytically inactive and oxidized Rh surfaces. A bulk Rh (Rh_{bulk}) and a surface component (Rh_s, chemical shift -0.6 eV) are observed for clean surfaces. Upon introducing O_2 and H_2, up to three additional components related to oxygen-bound Rh ($Rh_{O1/4}$, chemical shift -0.3 eV; $Rh_{O1/2}$, chemical shift +0.3 eV; $Rh_{O2/3}$, chemical shift +0.7 eV) may be present, depending on the surface coverage of oxygen (and thus state of catalytic activity), while the surface component disappears. In the notation $Rh_{Oi/j}$, i and j refer to the number of O atoms each Rh surface atom is bound to and the number of Rh surface atoms each O atom is bound to, respectively. All of these components have also previously been observed in studies of oxygen adsorption and H_2 oxidation on various Rh surfaces and were

corroborated by theoretical calculations *(S10–S12)*. Upon exposing a Rh surface to oxygen partial pressures in the 10^{-5} to 10^{-4} mbar range, Rh oxides can form, displaying a distinctive additional component (Rh_{oxide}, chemical shift +1.1 eV) in the Rh $3d_{5/2}$ spectra *(S13–S16)*. Binding energies/chemical shifts, FWHM, G/L ratio and asymmetry were considered to be identical in all spectra and optimized considering the whole ensemble of spectra and the corresponding O *1s* spectra in case of oxygen-related components (in order to assure correct Rh/O atomic ratios). FWHM, G/L ratio and asymmetry were assumed to be identical for all oxygen-related spectra components.

The Zr *3d* spectra were deconvoluted based on previous studies of the initial oxidation of Zr *(S17, S18)*: The clean bulk ZrO_2 support material was modeled using two symmetric Zr^{4+} components (Zr $3d_{5/2}$ and Zr $3d_{3/2}$), separated by 2.4 eV and having a fixed area ratio of 3:2. Binding energies, FWHM and G/L ratio were optimized using this spectrum. For convincingly reproducing all other Zr *3d* spectra, two additional doublets had to be included, corresponding to Zr^{3+} and Zr^{2+} sub-oxides. The binding energies/chemical shifts were optimized using all Zr *3d* spectra (Zr^{3+}: -0.9 eV, Zr^{2+}: -2.2 eV) and correspond well to the ones previously observed. FWHM and G/L ratio were assumed to be identical for all spectral components and for all recorded spectra.

Deconvolution of the O *1s* spectra started from the spectrum of the clean bulk ZrO_2 support, having a single symmetric component. In addition, a component related to oxygen adsorbed on Rh was introduced whenever Rh was present as per the Rh $3d_{5/2}$ spectra, in line with our previous observations of H_2 oxidation on Rh *(S8)*, while binding energy, FWHM and G/L ratio were considered to be identical for all recorded spectra and optimized considering the whole ensemble of spectra. In order to fully reproduce the Zr *3d* spectra whenever Zr sub-oxides were involved, a third component was necessary to be added to the spectra, corresponding to oxygen in the sub-oxides. Again, binding energy, FWHM, and G/L ratio were considered to be identical for all recorded spectra and optimized considering the whole ensemble of spectra.

To verify that the sample was in the same chemical state in the PEEM and SPEM experiments, and thus that the studies can be considered *correlative* despite being performed in different experimental setups, kinetic transition points (which can be detected by monitoring the $Rh_{O1/4}$ component in the Rh $3d_{5/2}$ spectra) were also probed by SPEM.

(S)TEM/EDX characterization

The (S)TEM/EDX characterization of the Rh/Au and Rh/ZrO_2 particles was performed at the University Service Center for Transmission Electron Microscopy (USTEM) at TU Wien after the PEEM and SPEM experiments. Cross-sections of the "big" particles were prepared by focused ion beam (FIB) milling (using Ga^+ ions at 30 keV) in a FEI Quanta 200 3D dual beam electron microscope. The cross-sections were investigated in a FEI Tecnai G²20 S-TWIN transmission electron microscope operated at 200 kV, which is equipped with a Gatan Orius 600 camera for imaging and diffraction imaging and an EDAX-AMETEK Octane T Elite plus detector for EDX.

Computational procedures

The mean-field micro-kinetic model applied in our simulations is based on the Langmuir-Hinshelwood mechanism, which is well established for H_2 oxidation on Rh surfaces *(S19)*, and the previously established mechanism of kinetic oscillations *(S8, S20–S22)*.

The reaction network is described by four equations (S1)-(S4), where $*$ denotes an empty surface site and $*_{sub}$ denotes an empty subsurface site:

$$O_2 + 2* \leftrightarrow O_{2,pre} + 2* \leftrightarrow 2O_{ad} \qquad \text{(Eq. S1)}$$

$$H_{ad} + 2* \leftrightarrow 2H_{ad} \qquad \text{(Eq. S2)}$$

$$O_{ad} + *_{sub} \leftrightarrow O_{sub} + * \qquad \text{(Eq. S3)}$$

$$2H_{ad} + O_{ad} \rightarrow H_{ad} + OH_{ad} + * \rightarrow H_2O + 3* \qquad \text{(Eq. S4)}$$

The formation of water takes place via an OH_{ad} intermediate, where the formation of the intermediate from O_{ad} and H_{ad} is the rate-limiting step in comparison to the subsequent reaction with a second H_{ad} species. Alternative ways of water formation (e.g., by OH_{ad} disproportioning) can be neglected *(S23)* and the reaction product water immediately desorbs at the present conditions *(S24)*. The coverages for oxygen θ_O, subsurface oxygen θ_S and hydrogen θ_H can then be described by three kinetic equations (S5)-(S7), where the empty sites θ_* are given by $\theta_* = 1 - \theta_H - \theta_O$.

$$\frac{\mathrm{d}\theta_{\mathrm{O}}}{\mathrm{d}t} = \frac{2}{1+K{\theta_*}^2}\left(k_{\mathrm{a}}^{\mathrm{O}} K p_{\mathrm{O}_2}{\theta_*}^2 - k_{\mathrm{d}}^{\mathrm{O}}{\theta_{\mathrm{O}}}^2\right) - k_{\mathrm{ox}}\theta_{\mathrm{O}}(1-\theta_{\mathrm{s}}) + k_{\mathrm{red}}\theta_{\mathrm{s}}\theta_* - k_{\mathrm{r}}\theta_{\mathrm{H}}\theta_{\mathrm{O}} \qquad \text{(Eq. S5)}$$

$$\frac{\mathrm{d}\theta_{\mathrm{s}}}{\mathrm{d}t} = k_{\mathrm{ox}}\theta_{\mathrm{O}}(1-\theta_{\mathrm{s}}) - k_{\mathrm{red}}\theta_{\mathrm{s}}\theta_* \qquad \text{(Eq. S6)}$$

$$\frac{\mathrm{d}\theta_{\mathrm{H}}}{\mathrm{d}t} = 2k_{\mathrm{a}}^{\mathrm{H}} p_{\mathrm{H}_2}{\theta_*}^2 - 2k_{\mathrm{d}}^{\mathrm{H}}{\theta_{\mathrm{H}}}^2 - 2k_{\mathrm{r}}\theta_{\mathrm{H}}\theta_{\mathrm{O}} \qquad \text{(Eq. S7)}$$

The rate constants in (S5)-(S7) are given by expressions (S8)-(S15), where $\beta = 1/k_B T$. The symbols are explained in Table S1.

$$k_{\mathrm{a}}^{\mathrm{H}} = S_0^{\mathrm{H}}\, a_{\mathrm{s}} / \sqrt{2\pi m_{\mathrm{H}_2} k_{\mathrm{B}} T} \qquad \text{(Eq. S8)}$$

$$k_{\mathrm{a}}^{\mathrm{O}} = S_0^{\mathrm{O}}\, a_{\mathrm{s}} / \sqrt{2\pi m_{\mathrm{O}_2} k_{\mathrm{B}} T} \qquad \text{(Eq. S9)}$$

$$k_{\mathrm{d}}^{\mathrm{H}} = k_{\mathrm{d0}}^{\mathrm{O}} e^{-\beta E_{\mathrm{d}}^{\mathrm{H}}} \qquad \text{(Eq. S10)}$$

$$K = K_0 e^{-\beta\left(E_{\mathrm{K}} + A_{\mathrm{K}}^{\mathrm{O}}\,\theta_{\mathrm{O}} + A_{\mathrm{K}}^{\mathrm{S}}\theta_{\mathrm{s}}\right)} \qquad \text{(Eq. S11)}$$

$$k_{\mathrm{d}}^{\mathrm{O}} = k_{\mathrm{d0}}^{\mathrm{O}} e^{-\beta\left(E_{\mathrm{d}}^{\mathrm{O}} + A_{\mathrm{d}}^{\mathrm{O}}\,\theta_{\mathrm{O}} + B_{\mathrm{d}}^{\mathrm{O}}\,{\theta_{\mathrm{O}}}^2\right)} \qquad \text{(Eq. S12)}$$

$$k_{\mathrm{ox}} = k_{\mathrm{ox}}^{0} e^{-\beta E_{\mathrm{ox}}} \qquad \text{(Eq. S13)}$$

$$k_{\mathrm{red}} = k_{\mathrm{red}}^{0} e^{-\beta\left(E_{\mathrm{red}} + A_{\mathrm{red}}^{\mathrm{S}}\theta_{\mathrm{s}}\right)} \qquad \text{(Eq. S14)}$$

$$k_{\mathrm{r}} = k_{\mathrm{r}}^{0} e^{-\beta\left(E_{\mathrm{r}} + A_{\mathrm{r}}^{\mathrm{H}}\theta_{\mathrm{H}} + A_{\mathrm{r}}^{\mathrm{O}}\theta_{\mathrm{O}}\right)} \qquad \text{(Eq. S15)}$$

In order to model the three different systems studied in the experiments (Rh/Rh, Rh/Au and Rh/ZrO_2), three parameter sets were used. The set for Rh/Rh is closely related to the one used for the step edge sites on Rh(110) in our recent studies on coexisting multi-states in H_2 oxidation on Rh *(S8)*, as the Rh particles are expected to expose surface structures of similar roughness. The set differs slightly only in the choice of S_0^O, A_K^S, A_{red}^s and E_d^H which have been adjusted to account for the particles exposing a mixture of several different surface structures.
For modeling the hinderance of dissociative hydrogen adsorption by the RhAu surface alloy and a resulting "smoothing" of the particle shape, S_0^H, E_{ox} and E_{red} were modified in the parameter set for the Rh/Au system. For modeling Rh/ZrO_2, E_d^O, A_K^O, A_K^S, E_{ox}, E_{red} and E_r were modified in comparison to the Rh/Rh parameter set, in line with the stronger binding of oxygen in the vicinity of the metal/oxide interface and a slight reduction of the surface roughness due to partially blocked step edges. All used model parameters are summarized in Table S1.
In addition to the already well-established simulations of the oscillating reaction mode at constant external parameters, in the present work kinetic transitions were modeled as well. For this, the p(H_2) parameter was ramped from 5.0×10^{-7} mbar to 2.0×10^{-5} mbar and back at constant T and p(O_2) parameters. The kinetic transition points were then extracted from the turnover frequency vs. p(H_2) data series by determining the respective inflection points.

Figures S1-S5

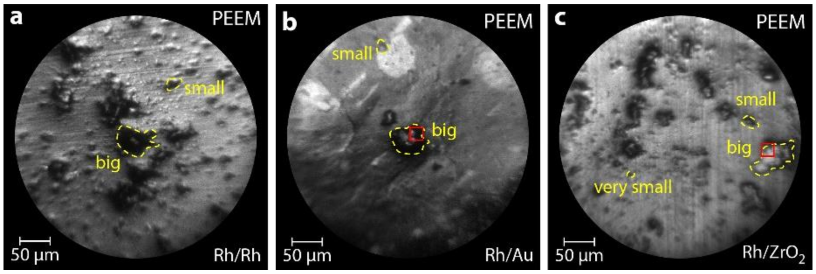

Figure S1. PEEM images of the clean Rh particles on the three different supporting materials with the "big" and "small" particles marked. **(a)** Rh/Rh; **(b)** Rh/Au; **(c)** Rh/ZrO_2. The red rectangles in (b) and (c) correspond to the areas imaged by SPEM in Figs. 3 and 4, correspondingly.

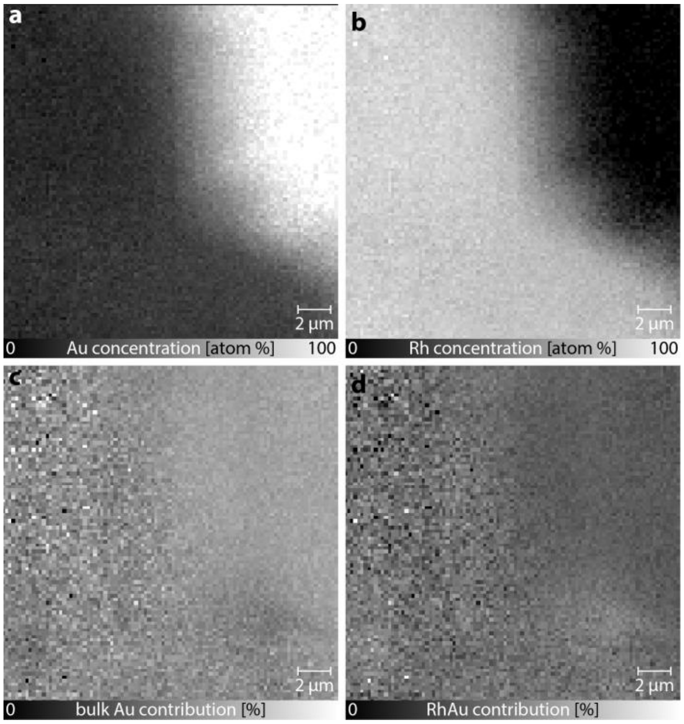

Figure S2. SPEM chemical maps of surface alloying on a Au supported Rh particle in the catalytically active state at T = 453 K, p(O_2) = 2.2 x 10^{-6} mbar, p(H_2) = 4.0 x 10^{-6} mbar. The maps depict the individual color-coded contributions in Figs. 3d and 3e. **(a)** atomic concentration of Au; **(b)** atomic concentration of Rh; **(c)** bulk Au contribution to the total Au $4f_{7/2}$ intensity; **(d)** RhAu surface alloy contribution to the total Au $4f_{7/2}$ intensity.

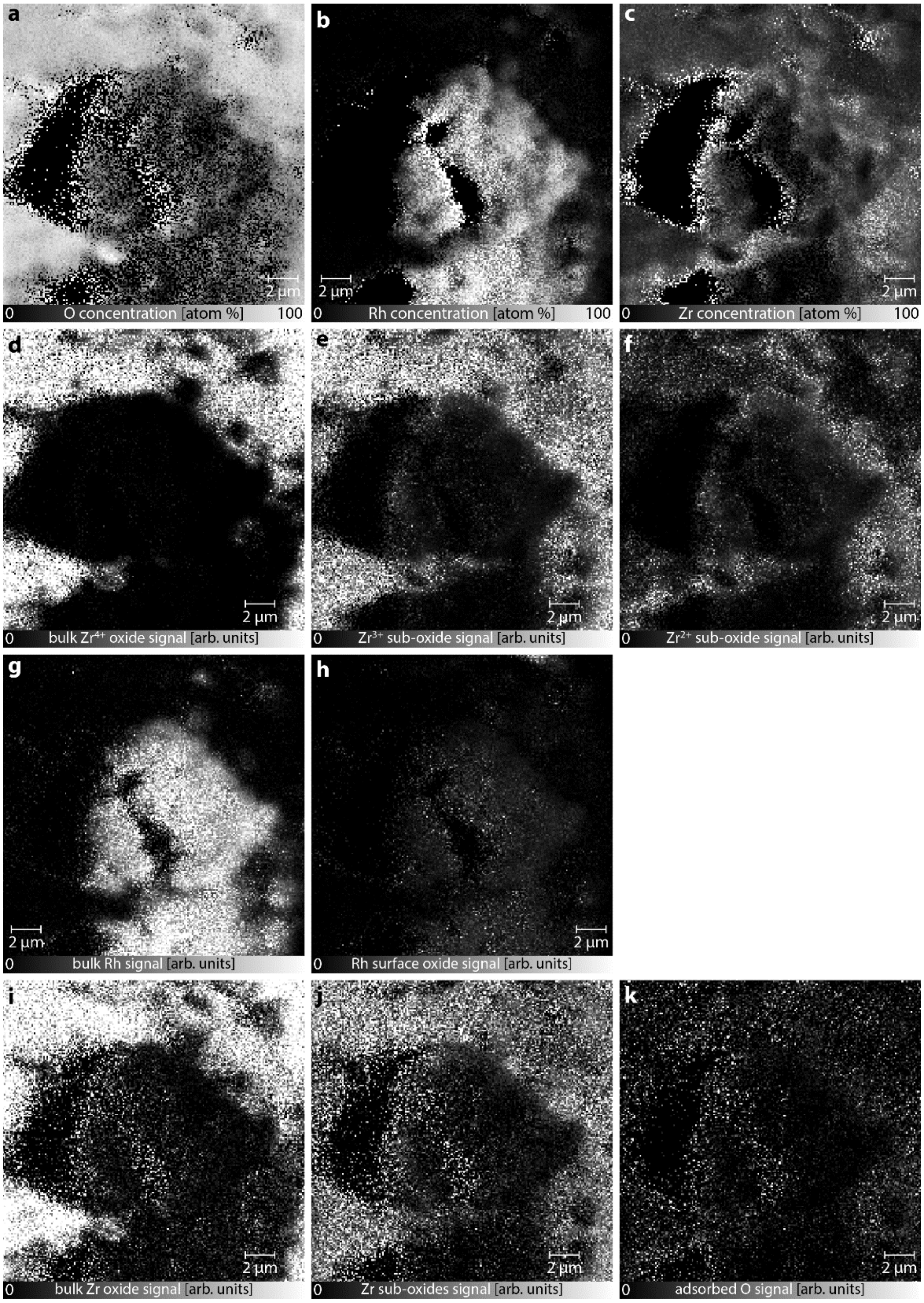

Figure S3. SPEM chemical maps of metal/oxide interface effects on ZrO_2 supported Rh particles in the catalytically active state at T = 453 K, p(O_2) = 2.2 x 10^{-6} mbar, p(H_2) = 4.0 x 10^{-6} mbar. The maps depict the individual color-coded contributions in Figs. 4d-g. **(a)** atomic concentration of O; **(b)** atomic concentration of Rh; **(c)** atomic concentration of Zr; **(d)** bulk Zr^{4+} oxide signal in the Zr *3d* map; **(e)** Zr^{3+} sub-oxide signal in the Zr *3d* map; **(f)** Zr^{2+} sub-oxide signal in the Zr *3d* map; **(g)** bulk Rh signal in the Rh $3d_{5/2}$ map; **(h)** Rh surface oxide signal in the Rh $3d_{5/2}$ map; **(i)** bulk Zr oxide in the O *1s* map; **(j)** Zr sub-oxides signal in the O *1s* map; **(k)** adsorbed O signal in the O *1s* map.

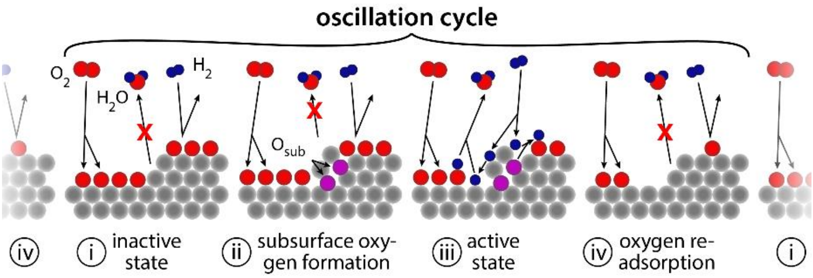

Figure S4. The oscillation cycle in catalytic H_2 oxidation on Rh. **(i)** the cycle starts from the catalytically inactive state, where preferential adsorption of oxygen at the step edges hinders dissociative adsorption of hydrogen and thus hampers catalytic activity; **(ii)** because of the dense oxygen coverage, oxygen starts to penetrate the Rh surface at kink or step sites. Some Rh atoms get dislocated, increasing the local surface roughness and creating favorable conditions for dissociative hydrogen adsorption; **(iii)** a switch to the catalytically active state takes place, where both hydrogen and oxygen adsorb, and then form water. Eventually, subsurface oxygen will diffuse to surface sites and react. Oxygen will once again be preferred at the step edges, resulting in a lack of hydrogen supply and the surface switching back to the catalytically inactive state; **(iv)** oxygen at the surface is replenished during the last stage and the cycle restarts.

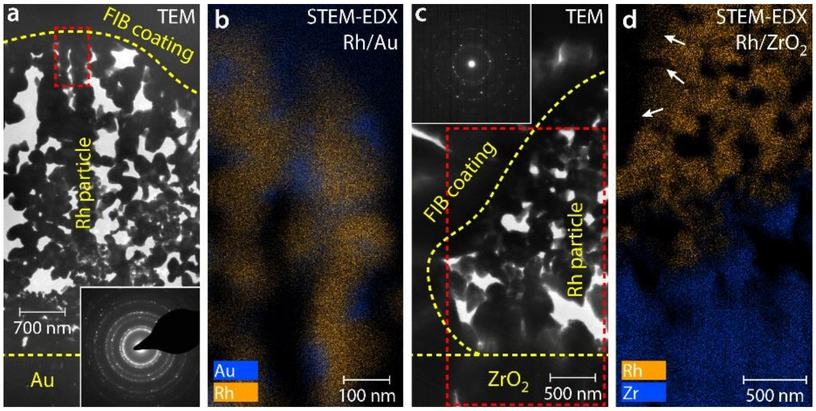

Figure S5. Transmission electron microscopy (TEM) and energy-dispersive X-ray fluorescence (EDX) of selected particles studied before by PEEM and SPEM. **(a)** cross-section TEM image of the “big” Rh particle on Au, prepared by focused ion beam (FIB). The yellow dashed lines indicate the boundaries of the Au support and the Rh particle. The inset shows a diffraction pattern obtained from within the Rh region, highlighting the polycrystalline nature of the Rh particle; **(b)** STEM-EDX compositional map of the area marked by the red dashed rectangle in (a), i.e., near the particle surface (blue: Au; orange: Rh), indicating decoration by Au; **(c)** cross-section TEM image of the metal/support boundary area of the “big” Rh particle on ZrO_2 prepared by FIB. The yellow dashed lines mark the boundaries of the ZrO_2 support and the Rh particle. The inset shows a diffraction pattern from within the Rh region; (**d)** STEM-EDX compositional map of the area indicated by the red dashed rectangle in (c), indicating ZrO_x species mainly near the metal/support boundary (orange: Rh, blue: Zr). Small oxide patches on Rh surface are marked by white arrows.

Tables S1-S2

Table S1. Parameter sets used in the micro-kinetic model simulations of the Rh/Rh, Rh/Au and Rh/ZrO_2 systems. Energies are given in eV, the area of a surface site in Å² and rate constants in 1/s. When no value is given, the same value as for Rh/Rh was used.

symbol	description	Rh/Rh	Rh/Au	Rh/ZrO_x
a_s	area of a surface site	10		
S_0^O	initial sticking coefficient of O	0.82		
k_{d0}^O	pre-factor for oxygen desorption	6.0 x 10^{13}		
E_d^O	desorption energy of O	3.20		3.70
A_d^O	dependence of oxygen desorption energy on adsorbed oxygen coverage	-0.5		
B_d^O	dependence of oxygen desorption energy on molecular oxygen coverage	-0.7		
K_0	pre-factor for oxygen dissociation equilibrium constant	0.2525		
E_k	activation energy for oxygen dissociation equilibrium constant	-0.178		
A_K^O	dependence of oxygen dissociation on adsorbed oxygen coverage	0.158		0.125
A_K^S	dependence of oxygen dissociation on sub-surface oxygen coverage	0.088		0.089
k_{ox}^0	pre-factor for oxygen diffusion from surface to sub-surface sites	5.0 x 10^{11}		
E_{ox}	activation energy for oxygen diffusion from surface to sub-surface sites	1.193	1.247	1.221
k_{red}^0	pre-factor for oxygen diffusion from sub-surface to surface sites	1.85 x 10^{13}		
E_{red}	activation energy for oxygen diffusion from sub-surface to surface sites	1.220	1.261	1.241
A_{red}^s	dependence of sub-surface oxygen reduction on sub-surface oxygen coverage	0.36		
S_0^H	initial sticking coefficient of H	0.40	0.37	
k_{d0}^h	pre-factor for hydrogen desorption	3.0 x 10^{10}		
E_d^H	desorption energy of H	0.75		
k_r^0	pre-factor for water formation	7.0 x 10^{12}		

Table S1. (continued)

symbol	description	Rh/Rh	Rh/Au	Rh/ZrOx
E_r	activation energy for water formation	0.79		0.80
A_r^H	dependence of activation energy of water formation on H coverage	-0.27		
A_r^O	dependence of activation energy of water formation on O coverage	-0.145		

Table S2. Energy windows used in constructing the chemical maps in Figs. 3 and 4.

	species	lower binding energy boundary [eV]	upper binding energy boundary [eV]
**Rh/Au: Au *4f*$_{7/2}$** (Fig. 3e)	Au bulk	84.40	85.39
	RhAu alloy	82.60	83.58
**Rh/ZrO$_2$: Zr *3d*** (Fig. 4e)	bulk Zr^{4+} oxide	181.30	182.18
	Zr^{3+} sub-oxide	180.43	180.96
	Zr^{2+} sub-oxide	179.15	180.03
**Rh/ZrO$_2$: Rh *3d*$_{5/2}$** (Fig. 4f)	Rh bulk	308.20	309.02
	Rh surface oxide	307.26	307.59
**Rh/ZrO$_2$: O *1s*** (Fig. 4g)	bulk Zr oxide	529.77	530.30
	Zr sub-oxides	528.18	528.71
	adsorbed O	526.95	527.65

References

(S*1*) Casalis, L.; Jark, W.; Kiskinova, M.; Lonza, D.; Melpignano, P.; Morris, D.; Rosei, R.; Savoia, A.; Abrami, A.; Fava, C.; Furlan, P.; Pugliese, R.; Vivoda, D.; Sandrin, G.; Wei, F. -Q.; Contarini, S.; DeAngelis, L.; Gariazzo, C.; Nataletti, P.; Morrison, G. R. ESCA Microscopy Beamline at ELETTRA. *Rev. Sci. Instrum.* **1995**, *66* (10), 4870–4875. DOI: 10.1063/1.1146167.

(S*2*) Gregoratti, L.; Barinov, A.; Benfatto, E.; Cautero, G.; Fava, C.; Lacovig, P.; Lonza, D.; Kiskinova, M.; Tommasini, R.; Mähl, S.; Heichler, W. 48-Channel Electron Detector for Photoemission Spectroscopy and Microscopy. *Rev. Sci. Instrum.* **2004**, *75* (1), 64–68. DOI: 10.1063/1.1630837.

(S*3*) Zeller, P.; Amati, M.; Sezen, H.; Scardamaglia, M.; Struzzi, C.; Bittencourt, C.; Lantz, G.; Hajlaoui, M.; Papalazarou, E.; Marino, M.; Fanetti, M.; Ambrosini, S.; Rubini, S.; Gregoratti, L. Scanning Photoelectron Spectro-Microscopy: A Modern Tool for the Study of Materials at the Nanoscale. *Phys. Status Solidi A* **2018**, *215* (19), 1800308. DOI: 10.1002/pssa.201800308.

(S*4*) Evans, S. Curve Synthesis and Optimization Procedures for X-Ray Photoelectron Spectroscopy. *Surf. Interface Anal.* **1991**, *17* (2), 85–93. DOI: 10.1002/sia.740170204.

(S*5*) Lytken, O. EccentricXPS - Analysis and Organisation of XPS Spectra https://www.wavemetrics.com/project/EccentricXPS.

(S*6*) Shirley, D. A. High-Resolution X-Ray Photoemission Spectrum of the Valence Bands of Gold. *Phys. Rev. B* **1972**, *5* (12), 4709–4714. DOI: 10.1103/PhysRevB.5.4709.

(S*7*) Óvári, L.; Berkó, A.; Vári, G.; Gubó, R.; Farkas, A. P.; Kónya, Z. The Growth and Thermal Properties of Au Deposited on Rh(111): Formation of an Ordered Surface Alloy. *Phys. Chem. Chem. Phys.* **2016**, *18* (36), 25230–25240. DOI: 10.1039/C6CP02128J.

(S*8*) Winkler, P.; Zeininger, J.; Raab, M.; Suchorski, Y.; Steiger-Thirsfeld, A.; Stöger-Pollach, M.; Amati, M.; Gregoratti, L.; Grönbeck, H.; Rupprechter, G. Coexisting Multi-States in Catalytic Hydrogen Oxidation on Rhodium. *Nat. Commun.* **2021**, *12* (1), 6517. DOI: 10.1038/s41467-021-26855-y.

(S*9*) Winkler, P.; Zeininger, J.; Suchorski, Y.; Stöger-Pollach, M.; Zeller, P.; Amati, M.; Gregoratti, L.; Rupprechter, G. How the Anisotropy of Surface Oxide Formation Influences the Transient Activity of a Surface Reaction. *Nat. Commun.* **2021**, *12* (1), 69. DOI: 10.1038/s41467-020-20377-9.

(S*10*) Ganduglia-Pirovano, M. V.; Scheffler, M.; Baraldi, A.; Lizzit, S.; Comelli, G.; Paolucci, G.; Rosei, R. Oxygen-Induced Rh $3d_{5/2}$ Surface Core-Level Shifts on Rh(111). *Phys. Rev. B* **2001**, *63* (20), 205415. DOI: 10.1103/PhysRevB.63.205415.

(S*11*) Baraldi, A.; Lizzit, S.; Comelli, G.; Kiskinova, M.; Rosei, R.; Honkala, K.; Nørskov, J. K. Spectroscopic Link between Adsorption Site Occupation and Local Surface Chemical Reactivity. *Phys. Rev. Lett.* **2004**, *93* (4), 046101. DOI: 10.1103/PhysRevLett.93.046101.

(S*12*) Bianchettin, L.; Baraldi, A.; de Gironcoli, S.; Vesselli, E.; Lizzit, S.; Comelli, G.; Rosei, R. Surface Core Level Shift: High Sensitive Probe to Oxygen-Induced Reconstruction of Rh(100). *J. Phys. Chem. C* **2009**, *113* (30), 13192–13198. DOI: 10.1021/jp901223d.

(S*13*) Gustafson, J.; Mikkelsen, A.; Borg, M.; Lundgren, E.; Köhler, L.; Kresse, G.; Schmid, M.; Varga, P.; Yuhara, J.; Torrelles, X.; Quirós, C.; Andersen, J. N. Self-Limited Growth of a Thin Oxide Layer on Rh(111). *Phys. Rev. Lett.* **2004**, *92* (12), 126102. DOI: 10.1103/PhysRevLett.92.126102.

(S*14*) Gustafson, J.; Mikkelsen, A.; Borg, M.; Andersen, J. N.; Lundgren, E.; Klein, C.; Hofer, W.; Schmid, M.; Varga, P.; Köhler, L.; Kresse, G.; Kasper, N.; Stierle, A.; Dosch, H. Structure of a Thin Oxide Film on Rh(100). *Phys. Rev. B* **2005**, *71* (11), 115442. DOI: 10.1103/PhysRevB.71.115442.

(S*15*) Dri, C.; Africh, C.; Esch, F.; Comelli, G.; Dubay, O.; Köhler, L.; Mittendorfer, F.; Kresse, G.; Dudin, P.; Kiskinova, M. Initial Oxidation of the Rh(110) Surface: Ordered Adsorption and Surface Oxide Structures. *J. Chem. Phys.* **2006**, *125* (9), 094701. DOI: 10.1063/1.2345058.

(S*16*) Blomberg, S.; Westerström, R.; Martin, N. M.; Lundgren, E.; Andersen, J. N.; Messing, M. E.; Gustafson, J. A High Pressure X-Ray Photoelectron Spectroscopy Study of Oxidation and Reduction of Rh(100) and Rh Nanoparticles. *Surf. Sci.* **2014**, *628*, 153–158. DOI: 10.1016/j.susc.2014.06.009.

(S*17*) Bespalov, I.; Datler, M.; Buhr, S.; Drachsel, W.; Rupprechter, G.; Suchorski, Y. Initial Stages of Oxide Formation on the Zr Surface at Low Oxygen Pressure: An in Situ FIM and XPS Study. *Ultramicroscopy* **2015**, *159*, 147–151. DOI: 10.1016/j.ultramic.2015.02.016.

(S*18*) Ma, W.; Herbert, F. W.; Senanayake, S. D.; Yildiz, B. Non-Equilibrium Oxidation States of Zirconium during Early Stages of Metal Oxidation. *Appl. Phys. Lett.* **2015**, *106* (10), 101603. DOI: 10.1063/1.4914180.

(S*19*) Zum Mallen, M. P.; Williams, W. R.; Schmidt, L. D. Steps in Hydrogen Oxidation on Rhodium: Hydroxyl Desorption at High Temperatures. *J. Phys. Chem.* **1993**, *97* (3), 625–632. DOI: 10.1021/j100105a016.

(S*20*) Suchorski, Y.; Datler, M.; Bespalov, I.; Zeininger, J.; Stöger-Pollach, M.; Bernardi, J.; Grönbeck, H.; Rupprechter, G. Visualizing Catalyst Heterogeneity by a Multifrequential Oscillating Reaction. *Nat. Commun.* **2018**, *9* (1), 600. DOI: 10.1038/s41467-018-03007-3.

(S*21*) Suchorski, Y.; Datler, M.; Bespalov, I.; Zeininger, J.; Stöger-Pollach, M.; Bernardi, J.; Grönbeck, H.; Rupprechter, G. Surface-Structure Libraries: Multifrequential Oscillations in Catalytic Hydrogen Oxidation on Rhodium. *J. Phys. Chem. C* **2019**, *123* (7), 4217–4227. DOI: 10.1021/acs.jpcc.8b11421.

(S*22*) Suchorski, Y.; Zeininger, J.; Buhr, S.; Raab, M.; Stöger-Pollach, M.; Bernardi, J.; Grönbeck, H.; Rupprechter, G. Resolving Multifrequential Oscillations and Nanoscale Interfacet Communication in Single-Particle Catalysis. *Science* **2021**, *372* (6548), 1314–1318. DOI: 10.1126/science.abf8107.

(S*23*) McEwen, J.-S.; Gaspard, P.; Visart de Bocarmé, T.; Kruse, N. Oscillations and Bistability in the Catalytic Formation of Water on Rhodium in High Electric Fields. *J. Phys. Chem. C* **2009**, *113* (39), 17045–17058. DOI: 10.1021/jp901975w.

(S*24*) Gregoratti, L.; Baraldi, A.; Dhanak, V. R.; Comelli, G.; Kiskinova, M.; Rosei, R. Structural Effects on Water Formation from Coadsorbed H + O on Rh(100). *Surf. Sci.* **1995**, *340* (3), 205–214. DOI: 10.1016/0039-6028(95)00695-8.
